# To Cut Short Whooping-Cough in Twenty-Four Hours

**Published:** 1893-12

**Authors:** 


TO CUT SHORT WHOOPING-COUGH IN TWENTY-FOUR HOURS.—*The Illustrated Medical Journal* says that Dr. Moncoro treats pertussis with a ten per cent solution of resorcin, by applying the solution every two hours to the periglotteal region with a throat brush. The application is made four or five times at each seance. The theory of the treatment is that the disease is due to micro-organism and affects primarily the larynx. Cultures of the micro-organisms have been destroyed by the smallest amount of resorcin.—*Texas Sanitarian.*

